# Supplementary material for: A high-resolution mRNA expression time course of embryonic development in zebrafish
Source: eLife. 2017 Nov 16;6:e30860. doi: 10.7554/eLife.30860 (PMC5690287; doi:10.7554/eLife.30860)
Supplement: Supplementary file 6. [file elife-30860-supp6.zip › biolayout-clusters-files/Cluster144-genes.html]

Cluster144


# Cluster144: Genes

| Ensembl ID | Gene Name | Chr | Start | End | Biotype |
| --- | --- | --- | --- | --- | --- |
| ENSDARG00000099541 | DEPDC1B | KN150240.1 | 21351 | 28237 | protein\_coding |
| ENSDARG00000016775 | aqr | 17 | 50930341 | 51032977 | protein\_coding |
| ENSDARG00000045989 | arf4a | 11 | 42203969 | 42212378 | protein\_coding |
| ENSDARG00000052402 | dnmt3ba | 8 | 23334975 | 23359960 | protein\_coding |
| ENSDARG00000076815 | eif3s10 | 13 | 19152954 | 19186587 | protein\_coding |
| ENSDARG00000020595 | gnl3l | 11 | 25248158 | 25264875 | protein\_coding |
| ENSDARG00000006837 | mycn | 20 | 33391549 | 33396505 | protein\_coding |
| ENSDARG00000019529 | parp1 | 20 | 43751360 | 43792701 | protein\_coding |
| ENSDARG00000101627 | ssr1 | 24 | 42111928 | 42120840 | protein\_coding |
